# Natriuretic peptides to differentiate constrictive pericarditis and restrictive cardiomyopathy: A systematic review and meta‐analysis

**DOI:** 10.1002/clc.23772

**Published:** 2021-12-30

**Authors:** Carlos Diaz‐Arocutipa, Jose Saucedo‐Chinchay, Massimo Imazio, Edgar Argulian

**Affiliations:** ^1^ Vicerrectorado de Investigación Universidad San Ignacio de Loyola Lima Peru; ^2^ Programa de Atención Domiciliaria EsSalud Lima Peru; ^3^ Asociación para el Desarrollo de la Investigación Estudiantil en Ciencias de la Salud (ADIECS) Lima Peru; ^4^ Department of Emergency Hospital Nacional Edgardo Rebagliati Martins Lima Peru; ^5^ Cardiothoracic Department University Hospital “Santa Maria della Misericordia” Udine Italy; ^6^ Mount Sinai Heart Icahn School of Medicine at Mount Sinai New York USA

**Keywords:** constrictive pericarditis, natriuretic peptides, restrictive cardiomyopathy, systematic review

## Abstract

Previous studies have shown that natriuretic peptide levels are increased in patients with restrictive cardiomyopathy (RCM) but not in constrictive pericarditis (CP). We performed a systematic review and meta‐analysis to evaluate the diagnostic utility of B‐type natriuretic peptide (BNP) and N‐terminal pro‐brain natriuretic peptide (NT‐proBNP) to differentiate CP and RCM. We searched electronic databases from inception to January 07, 2021. Studies involving adult patients that assessed the utility of natriuretic peptides to differentiate CP and RCM were included. All meta‐analyses were performed using a random‐effects model. Seven studies (four case‐control and three cohorts) involving 204 patients were included. The mean age ranged between 25.7 and 64.1 years and 77% of patients were men. BNP levels were significantly lower (standardized median difference [SMD], −1.48; 95% confidence interval [CI], −2.33 to −0.63) in patients with CP compared to RCM. The pooled area under the curve (AUC) of the BNP level was 0.81 (95% CI, 0.70–0.92). NT‐proBNP (SMD, −0.86; 95% CI, −1.38 to −0.33) and log NT‐proBNP (SMD, −1.89; 95% CI, −2.59 to −1.20) levels were significantly lower in patients with CP compared to RCM. Our review shows that BNP and NT‐proBNP levels were significantly lower in patients with CP compared to RCM. The pooled AUC of BNP level showed a good diagnostic accuracy to differentiate both conditions.

## 1 INTRODUCTION

Constrictive pericarditis (CP) results from a chronic inflammatory process of the pericardium leading to a noncompliant, fibrotic, and/or calcified pericardium.^1^ This condition still represents a diagnostic challenge, being restrictive cardiomyopathy (RCM) considered as one of its most important differential diagnoses.^2^ The differentiation between CP and RCM is crucial because of their treatment and prognostic differences.^1^

Previous studies have shown that natriuretic peptides are elevated in RCM but not in CP.^3^, ^4^ However, this laboratory test is not routinely used in the assessment of these conditions.^5^ Therefore, we performed a systematic review and meta‐analysis to evaluate the diagnostic utility of B‐type natriuretic peptide (BNP) and N‐terminal pro‐brain natriuretic peptide (NT‐proBNP) to differentiate patients with CP and RCM.

## 2 METHODS

This review was reported according to the 2009 PRISMA (Preferred Reporting Items for Systematic Reviews and Meta‐Analyses) statement.^6^

### 2.1 Search strategy

We searched in the following electronic databases: PubMed, Embase, Scopus, and Web of Science. The search was conducted from inception to January 07, 2021. The complete search strategy is available in Table S1. There were no restrictions on language or publication date. Additionally, we conducted a hand‐searching of reference lists of all included studies and relevant reviews to identify further studies.

### 2.2 Eligibility criteria

Studies involving adult patients (≥18 years old) that evaluated the utility of natriuretic peptides to differentiate CP and RCM were included. We excluded conference abstracts, animal studies, editorials, commentaries, systematic reviews, and narrative reviews.

### 2.3 Study selection

We downloaded all articles from electronic search to EndNote X8 software and duplicate records were removed. Titles and abstracts were independently screened by two review authors (CDA and JSC) to identify relevant studies. The same review authors (CDA and JSC) independently evaluated the full text of the articles. Any disagreement on title/abstract and full‐text selection was resolved through consensus.

### 2.4 Data extraction

The data from each study were independently extracted by two review authors (CDA and JSC) using standardized data extraction and any disagreement was resolved through consensus. If additional data was needed, the corresponding author was contacted through email. We extracted the following information: first author name, year of publication, country, study design, population, sample size, age, sex, etiology, and diagnosis of CP and RCM, and natriuretic peptide levels.

### 2.5 Risk of bias assessment

The Quality Assessment of Diagnostic Accuracy Studies 2 (QUADAS‐2) tool was used to evaluate the quality of diagnostic accuracy studies.^7^ This tool includes the evaluation of the risk of bias (four domains) and concerns about applicability (three domains). Each domain will be judged as “low,” “high,” or “unclear.” The risk of bias of case‐control and cohort studies was assessed using the Newcastle‐Ottawa Scale (NOS) tool.^8^ Each study was classified as low risk of bias (8–9 points), moderate risk of bias (5–7 points), and high risk of bias (0–4 points). Two review authors (CDA and JSC) independently perform assessments and any disagreement was resolved by consensus.

### 2.6 Statistical analysis

For diagnostic accuracy studies, when individual patient data were available, we displayed a receiver operating characteristic (ROC) curve for each study. In addition, we used a random‐effects model to calculate the pooled area under the curve (AUC) with their 95% confidence interval (CI) of BNP level. For case‐control studies, the Dersimonian‐Laird random‐effects model was performed to pool standardized mean difference (SMD) with their 95% CIs of BNP, NT‐proBNP, and log NT‐proBNP levels. The SMD was chosen because there was variation in the type of assay used for the measurement of natriuretic peptides. Log NT‐proBNP represents the logarithmic transformation of the NT‐proBNP levels reported in the studies. We pooled NT‐proBNP and log NT‐proBNP separately because some studies only reported log NT‐proBNP and it was not possible to convert to NT‐proBNP. Heterogeneity among studies was evaluated using the *χ* ^2^ test (threshold *p* < .10) and *I*² statistic. Heterogeneity was defined as low if *I* ^2^ < 30%, moderate if *I* ^2^ = 30%–60%, and high if *I*² > 60%. Meta‐analyses were conducted using the software R 3.6.3 and the web IPD Meta‐Analysis of Diagnostic Accuracy.^9^ A two‐tailed *p* < .05 was considered statistically significant.

## 3 RESULTS

### 3.1 Study selection

**Figure 1:**
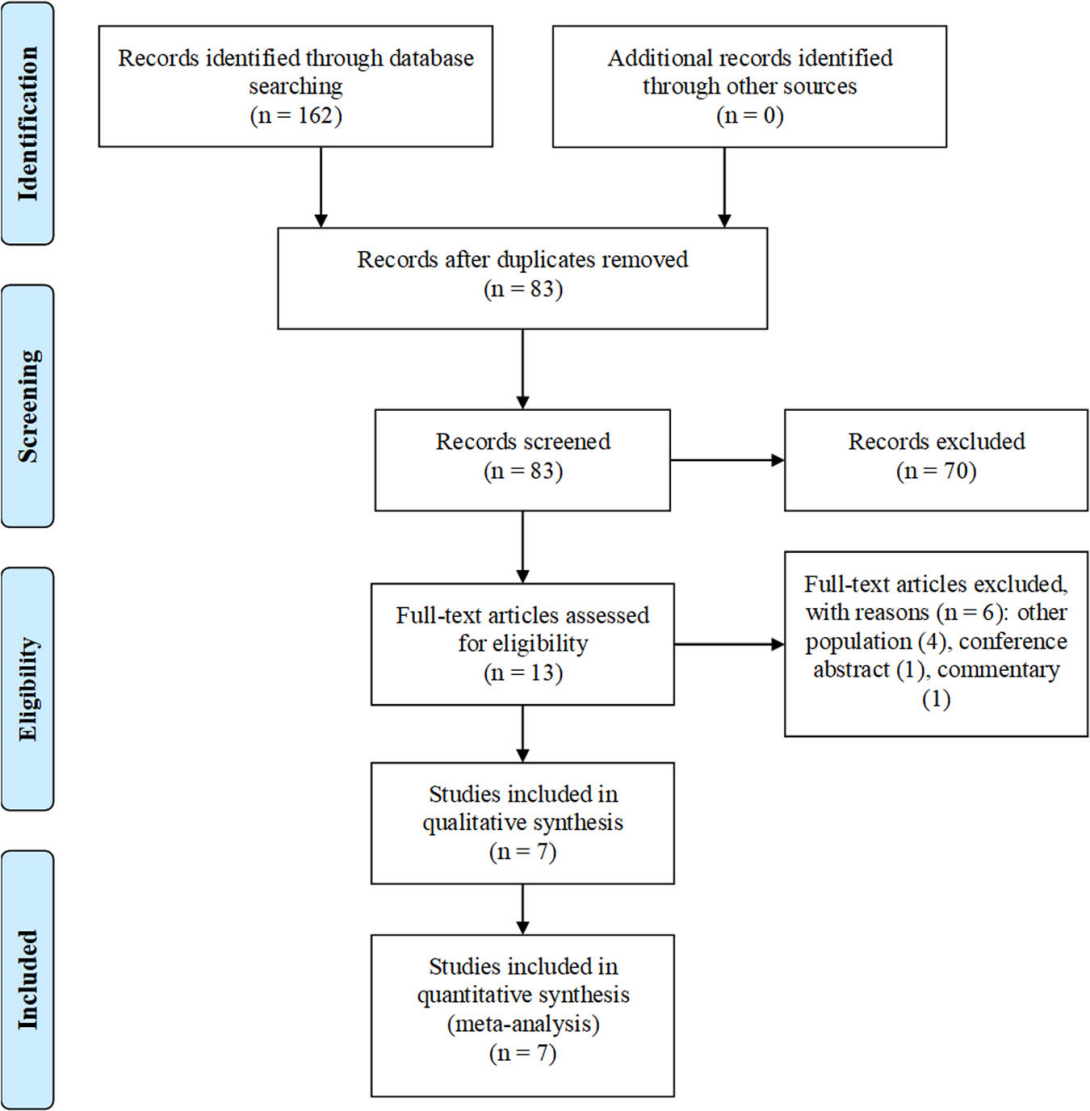
Flow diagram of study selection

Our electronic search retrieved 162 articles. After the removal of duplicates, 83 articles were screened by title/abstract, and of those, 70 were excluded. After a full‐text assessment of 13 remaining articles, six were excluded due to other populations (4), conference abstract (1), and commentary (1). Finally, seven articles^3^, ^4^, ^10^, ^11^, ^12^, ^13^, ^14^ were selected (Figure 1).

### 3.2 Study characteristics

The characteristics of the seven included studies (*n* = 204) are shown in Table 1. Four studies had a case‐control design and three studies were cohorts. Seventy‐seven percent of patients were men and the mean age ranged between 25.7 and 64.1 years. Four studies were conducted in the United States of America.

**Table 1:** Characteristics of included studies

Study	Design	Country	Population	CP diagnosis	Etiology of CP	RCM diagnosis	Etiology of RCM	Assay of natriuretic peptides	Group	Sample size	Age (years)^a^	Male (%)
Babuin et al. (2006)^3^	Case‐control	USA	Patients diagnosed with CP and RCM	Surgery	Idiopathic (11), cardiac surgery (8), and radiotherapy (3)	Echocardiography and cardiac catheterization	NR	BNP (Biosite assay)	CP	22	56.8 ± 10.7	77
									RCM	11	58.8 ± 10.2	54
Sengupta et al. (2008)^14^	Case‐control	USA	Patients diagnosed with CP and RCM	Surgery	Idiopathic (7), cardiac surgery (7), and radiotherapy (2)	Endomyocardial biopsy + echocardiography	Cardiac amyloidosis (15)	BNP (Biosite assay)	CP	16	61.8 ± 13	81
									RCM	15	60.5 ± 9	67
Karaahmet et al. (2009)^10^	Case‐control	Turkey	Patients with chronic symptoms of either CP or RCM	Surgery	Idiopathic (15), tuberculosis (1), and cardiac surgery (1)	Endomyocardial biopsy (6) + cardiac catheterization (6)	Idiopathic (6)	NT‐proBNP (Roche Diagnostics)	CP	17	34.4 ± 15.3	76
									RCM	8	36 ± 21.2	62
Parakh et al. (2015)^12^	Case‐control	India	Patients diagnosed with CP and RCM	Surgery (26) and other (3)	Tuberculosis (29)	Clinical features, echocardiography, CT, CMR, and cardiac catheterization	Cardiac amyloidosis (2), hemochromatosis (1), endomyocardial fibrosis (4), and idiopathic (13)	NT‐proBNP (Roche Diagnostics)	CP	29	25.7 ± 13.2	100
									RCM	20	39.2 ± 20	100
Leya et al. (2005)^4^	Cohort	USA	Patients with NYHA III or IV undergoing invasive hemodinamic assessment for evaluation of CP or RCM	Cardiac catheterization	NR	Cardiac catheterization	NR	BNP (ADVIA Centaur system)	CP	6	64.1 ± 11.2	67
									RCM	5	54.2 ± 19.8	40
Reddy et al. (2007)^13^	Cohort	USA	Consecutive patients with suspected CP or RCM	Cardiac catheterization or surgery	Thoracic surgery (13) and idiopathic (4)	Cardiac catheterization	NR	BNP (ADVIA Centaur system)	CP	17	60.2 ± 14.5	76
									RCM	5	NR	NR
Mady et al. (2008)^11^	Cohort	Brasil	Consecutive patients undergoing evaluation in the cardiomyopathy group	Surgery	Tuberculosis (2) and idiopathic (14)	Echocardiography	Endomyocardial fibrosis	NT‐proBNP (Roche Diagnostics)	CP	16	32 ± 16	56
									RCM	17	49 ± 9	18

Abbreviations: BNP, B‐type natriuretic peptide; CMR, cardiac magnetic resonance; CP, constrictive pericarditis; CT, computed tomography; NR, not reported; NT‐proBNP, N‐terminal pro‐brain natriuretic peptide; NYHA, New York Heart Association; RCM, restrictive cardiomyopathy.

^a^ Data are mean ± standard deviation.

In five studies, the diagnosis of CP was defined on the basis of surgical findings, while in the rest, it was made by cardiac catheterization. The most common etiologies of CP were idiopathic (49%), postcardiac surgery (27%), and tuberculosis (20%) across six studies. In two studies, the diagnosis of RCM was defined according to cardiac catheterization findings. In rest of the studies, it was based on echocardiography and/or endomyocardial biopsy. The etiology of RCM was reported in three studies. Endomyocardial fibrosis (40%) and cardiac amyloidosis (37%) were the most frequent etiological diagnosis. Only one study reported data on the impact of kidney function on natriuretic peptide levels.^13^ The type of assay used for BNP measurement was the Biosite BNP assay in two studies and the ADVIA Centaur BNP assay in two studies. In three studies, the assay used for NT‐proBNP was the Roche Diagnostics assay. Overall, four studies reported data on BNP, two studies on NT‐proBNP, and two studies on log NT‐proBNP. No studies reported information on log BNP.

### 3.3 Risk of bias assessment

The QUADAS‐2 scores for the risk of bias and applicability concerns are shown in Figure S1. Overall, the risk of bias was high or unclear for almost all domains. In contrast, concerns regarding applicability were generally low for most studies. The NOS tool for case‐control and cohort studies showed a moderate risk of bias for all studies (Tables S2 and S3).

### 3.4 BNP levels

**Figure 2:**
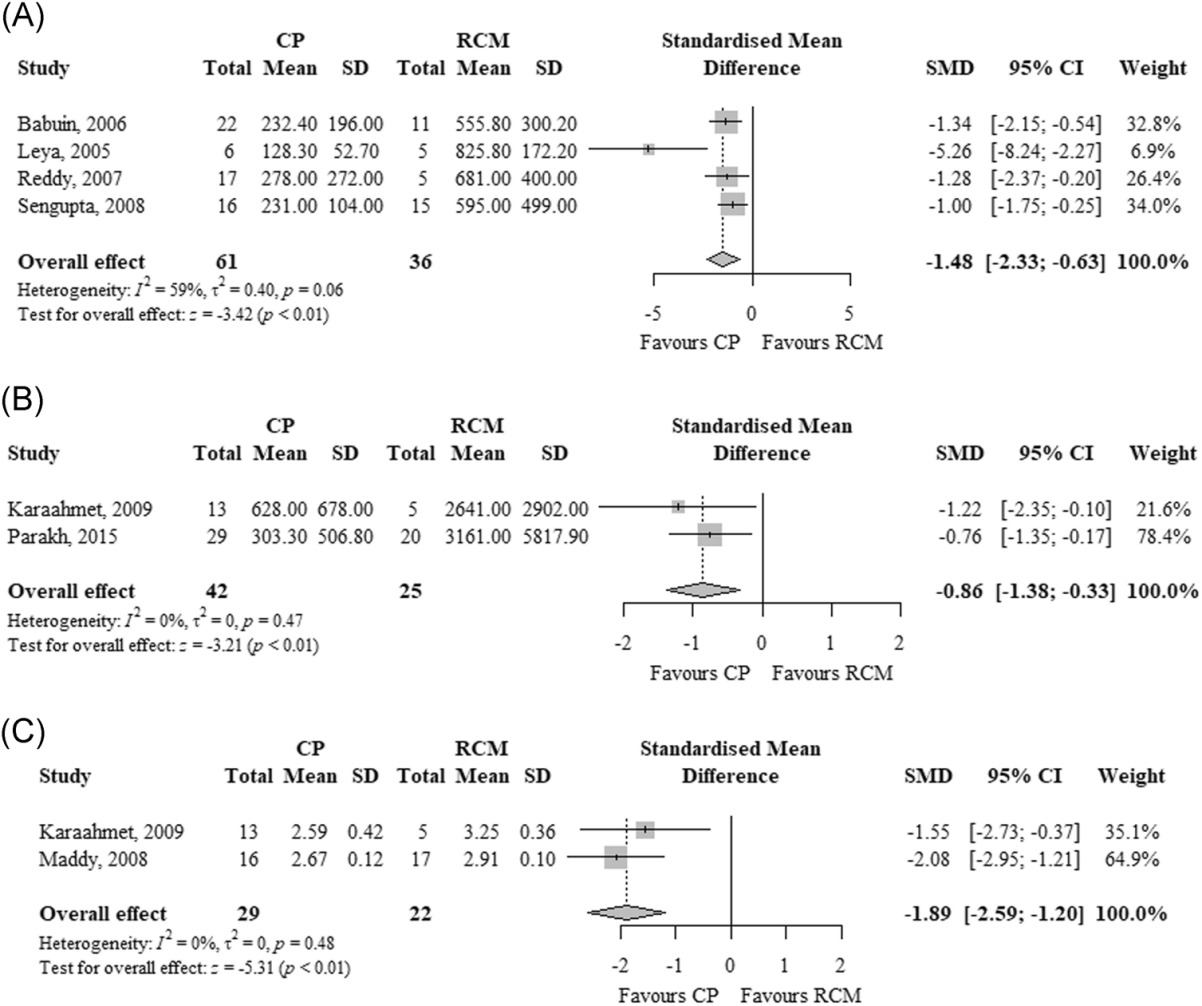
Forest plot showing the standardized mean difference of (A) BNP, (B) NT‐proBNP, and (C) log NT‐proBNP levels between patients with constrictive pericarditis and restrictive cardiomyopathy. BNP, B‐type natriuretic peptide; CI, confidence interval; NT‐proBNP, N‐terminal pro‐brain natriuretic peptide; RCM, restrictive cardiomyopathy; SMD, standardized mean difference

**Figure 3:**
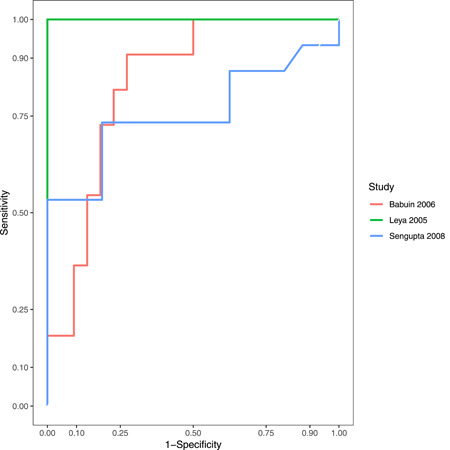
The receiver operating characteristic curves of the B‐type natriuretic peptide level to differentiate patients with constrictive pericarditis and restrictive cardiomyopathy

**Figure 4:**
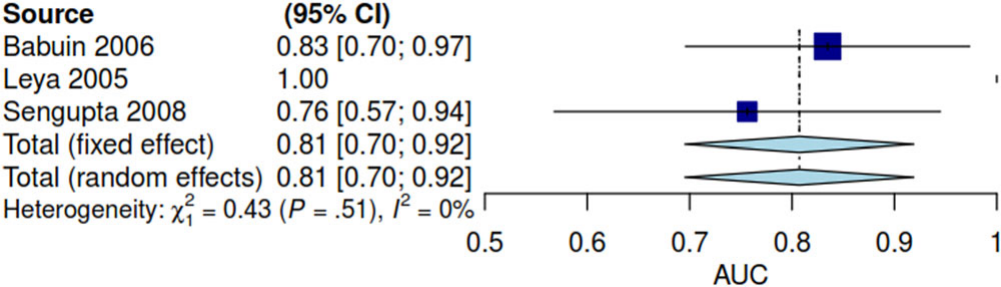
Forest plot showing of the AUC of BNP levels to differentiate patients with constrictive pericarditis and restrictive cardiomyopathy. AUC, area under the curve; BNP, B‐type natriuretic peptide; CI, confidence interval

In four studies (two case‐control and two cohorts, *n* = 97), the BNP levels were significantly lower (SMD, −1.48; 95% CI, −2.33 to −0.63; *I* ^2^ = 59%) in patients with CP compared to RCM (Figure 2). In three studies with individual patient data, the ROC curves are shown in Figure 3. The pooled AUC of BNP level was 0.81 (95% CI, 0.70–0.92) (Figure 4). Given the heterogeneity of the included studies, we have not estimated pooled sensitivity and specificity at a specific cut‐off point.

### 3.5 NT‐proBNP levels

In two studies (two case‐control, *n* = 67), the NT‐proBNP levels were significantly lower (SMD, −0.86; 95% CI, −1.38 to −0.33; *I* ^2^ = 0%) in patients with CP compared to RCM (Figure 2). Likewise, in two studies (one case‐control and one cohort, *n* = 51), the log NT‐proBNP levels were significantly lower (SMD, −1.89; 95% CI, −2.59 to −1.20; *I* ^2^ = 0%) in patients with CP compared to RCM (Figure 2). The ROC curves using NT‐proBNP or log NT‐proBNP were not available due to lack of data.

## 4 DISCUSSION

We found that plasma concentrations of BNP and NT‐proBNP were significantly lower in patients with CP compared to RCM. The pooled AUC of BNP level showed an adequate diagnostic performance to differentiate both conditions. However, the risk of bias was moderate or high across studies.

Patients with CP and RCM share similar clinical and hemodynamic features.^1^ Thus, the distinction between these two conditions can be challenging, especially during the assessment of patients with an initial diagnosis of heart failure with preserved ejection fraction.^5^ Diagnostic criteria of CP rely primarily on two‐dimensional echocardiography and Doppler findings, and multimodality imaging (including computed tomography and cardiac magnetic resonance.^5^ Indeed, in some cases, a definitive diagnosis can only be made by invasive tools (cardiac catheterization and cardiac surgery).

Natriuretic peptides are widely used biomarkers in routine clinical practice and have an established diagnostic and prognostic role in patients with heart failure.^15^ In addition, natriuretic peptides have also been shown to be useful in other cardiac diseases such as valvular heart disease, coronary artery disease, and cardiomyopathies, particularly RCM, which is characterized by substantial diastolic dysfunction due to intrinsic myocardial disease.^15^ In contrast, although CP is also characterized by abnormal ventricular diastolic filling, it is primarily caused by a disease of the pericardium not involving the myocardium unless in mixed forms or advanced cases.^2^ Given that the myocardium is usually normal in CP and the myocardial stretch is impeded by the pericardial constraint,^4^ it has been suggested that measurement of natriuretic peptides could be valuable in the diagnostic work‐up to differentiate constrictive from restrictive physiology.^16^ On the basis of our results, CP should be suspected when normal or slightly increased levels of natriuretic peptides are detected in patients with unexplained heart failure, especially when other physical and echocardiographic findings coexist. However, more studies with larger sample sizes are still needed to confirm the diagnostic utility of natriuretic peptides in CP.

BNP and NT‐proBNP are secreted by cardiomyocytes predominantly in response to wall stress.^15^ Although plasma levels of BNP and NT‐proBNP are similar in normal individuals, NT‐proBNP rises more than BNP in patients with heart failure.^17^ We found that the levels of the two natriuretic peptides are significantly reduced in patients with CP in comparison to RCM; therefore, both biomarkers seem to be equally useful, although BNP was the most studied. On the contrary, there is some variation in their values according to the following patient's characteristics: age, sex, body mass index, and kidney function.^17^ Accordingly, plasma concentrations of natriuretic peptides tend to increase with age, to be higher in women, lower in obese people, and higher in patients with kidney dysfunction. Unfortunately, only one study provided data on kidney function. Reddy et al.^13^ reported that patients with CP and glomerular filtration rate <90 ml/min had similar BNP levels as RCM and were significantly lower than patients with normal kidney function. Therefore, a higher diagnostic threshold may be required for patients with chronic kidney disease.

Currently, the etiology of CP has been characterized by a decrease in the frequency of idiopathic or viral causes and an increase of secondary causes such as postcardiac surgery, postpercutaneous cardiac interventions, postradiation therapy, among others.^1^ Plasma concentrations of natriuretic peptides appear to be related to the etiology of CP. Two previous studies^3^, ^14^ reported that patients with idiopathic CP had lower BNP levels compared with secondary causes, which was mainly related to prior cardiac surgery and radiotherapy. Furthermore, there was no significant difference in BNP levels between patients with secondary CP and RCM. Although in most cases of idiopathic CP the clinical presentation is dominated by the classic constrictive physiology, it is well recognized that many patients also have a previous history of myocardial disease.^18^ Therefore, it makes sense that natriuretic peptide levels are higher in patients with CP due to secondary causes compared to idiopathic cases.^19^ Therefore, the use of natriuretic peptides may not be useful in patients with non‐idiopathic forms of CP, and other imaging techniques such as cardiac computed tomography and cardiac magnetic resonance may be required. Nevertheless, this finding should be verified in future studies.

To our knowledge, this is the first systematic review that assessed the diagnostic utility of natriuretic peptides to differentiate CP and RCM. However, our study has some limitations. First, given that two different assay types were used for the measurement of BNP levels, it is possible that this could have influenced the estimates of diagnostic accuracy of BNP. Thus, these values should be considered exploratory only. Second, the gold standard for the CP and RCM diagnosis varied across studies. However, the diagnostic yield of these methods is reasonably adequate to diagnose these disorders. Third, the effect of other factors (age, obesity, atrial fibrillation, and diuretic therapy) that could affect the determination of natriuretic peptides have not been adequately evaluated in the included studies. Fourth, women were underrepresented across included studies. Thus, our results may not generalizable to the female population. Fifth, heterogeneity was moderate for pooled BNP levels across studies. Finally, given that our study included a small sample size for BNP and NT‐proBNP assays, limiting the generalizability of our findings.

## 5 CONCLUSIONS

Our review shows that plasma concentrations of BNP and NT‐proBNP were significantly lower in patients with CP compared to RCM. The pooled AUC of BNP level showed a good diagnostic accuracy to differentiate both conditions. However, further studies with a larger sample size using standardized measurements of natriuretic peptides are required to confirm our results.

## CONFLICT OF INTERESTS

The authors declare that there are no conflict of interests.

## AUTHOR CONTRIBUTIONS

Carlos Diaz‐Arocutipa and Jose Saucedo‐Chinchay were involved in concept/design and in data acquisition. Carlos Diaz‐Arocutipa, Jose Saucedo‐Chinchay, Massimo Imazio, and Edgar Argulian were involved in data analysis/interpretation. Carlos Diaz‐Arocutipa drafted the article. Jose Saucedo‐Chinchay, Massimo Imazio, and Edgar Argulian critically revised the article. Carlos Diaz‐Arocutipa, Jose Saucedo‐Chinchay, Massimo Imazio, and Edgar Argulian approved the article.

## Supporting information

Supporting information.

## Data Availability

The data that support the findings of this study are available from the corresponding author upon reasonable request.
