# Supplementary material for: Natriuretic peptides to differentiate constrictive pericarditis and restrictive cardiomyopathy: A systematic review and meta‐analysis
Source: Clin Cardiol. 2021 Dec 30;45(3):251–7. doi: 10.1002/clc.23772 (PMC8922532; doi:10.1002/clc.23772)
Supplement: Supplementary file 1 — Supporting information. [file CLC-45-251-s001.doc]

**SUPPLEMENTARY DATA**

**Table S1. Search strategy (January 07, 2021)**

| **PubMed (23 hits)**  (“pericarditis, constrictive”[mesh] OR “constrictive pericarditis”[tiab] OR constriction[tiab]) AND (“cardiomyopathy, restrictive”[mesh] OR restrictive[tiab] OR restriction[tiab]) AND (“natriuretic peptides”[mesh] OR “natriuretic peptide, brain”[mesh] OR “natriuretic peptide”[tiab] OR “natriuretic peptides”[tiab] OR BNP[tiab] OR “NT-proBNP”[tiab]) |
| --- |
| **Embase (65 hits)**  ('constrictive pericarditis'/exp OR 'constrictive pericarditis' OR 'constriction'/exp OR 'constriction') AND ('restrictive' OR 'restriction') AND ('natriuretic peptide'/exp OR 'natriuretic peptide' OR 'natriuretic peptides'/exp OR 'natriuretic peptides' OR 'bnp' OR 'nt-probnp') |
| **Scopus (226 hits)**  TITLE-ABS-KEY ((“constrictive pericarditis” OR “constriction”) AND (“restrictive” OR “restriction”) AND (“natriuretic peptide” OR “natriuretic peptides” OR “BNP” OR “NT-proBNP”)) |
| **Web of Science (32 hits)**  ALL=((“constrictive pericarditis” OR “constriction”) AND (“restrictive” OR “restriction”) AND (“natriuretic peptide” OR “natriuretic peptides” OR “BNP” OR “NT-proBNP”)) |

**Table S2. Newcastle-Ottawa scale for risk of bias assessment of case-control studies**

| **Study** | **SELECTION** | | | | **COMPARABILITY** | **EXPOSURE** | | | **Total (maximum = 9)** |
| --- | --- | --- | --- | --- | --- | --- | --- | --- | --- |
|  | **Is the case definition adequate** | **Representativeness of the cases** | **Selection of Controls** | **Definition of Controls** |  | **Ascertainment of exposure** | **Same method of ascertainment for cases and controls** | **Non-Response rate** |  |
| Karaahmet, 2009 | * | - | * | * | - | * | * | * | 6 |
| Parakh, 2015 | * | - | * | - | - | * | * | * | 5 |

**Table S3. Newcastle-Ottawa scale for risk of bias assessment of cohort studies**

| **Study** | **SELECTION** | | | | **COMPARABILITY** | **OUTCOME** | | | **Total (maximum = 9)** |
| --- | --- | --- | --- | --- | --- | --- | --- | --- | --- |
|  | **Representativeness of the exposed cohort** | **Selection of the non-exposed cohort** | **Ascertainment of the exposure** | **Outcome status at start of study** |  | **Assessment of the outcome** | **Length of follow-up** | **Adequacy of follow-up** |  |
| Reddy, 2007 | - | * | * | * | - | - | * | * | 5 |
| Mady, 2008 | - | * | * | * | - | * | * | * | 6 |

**
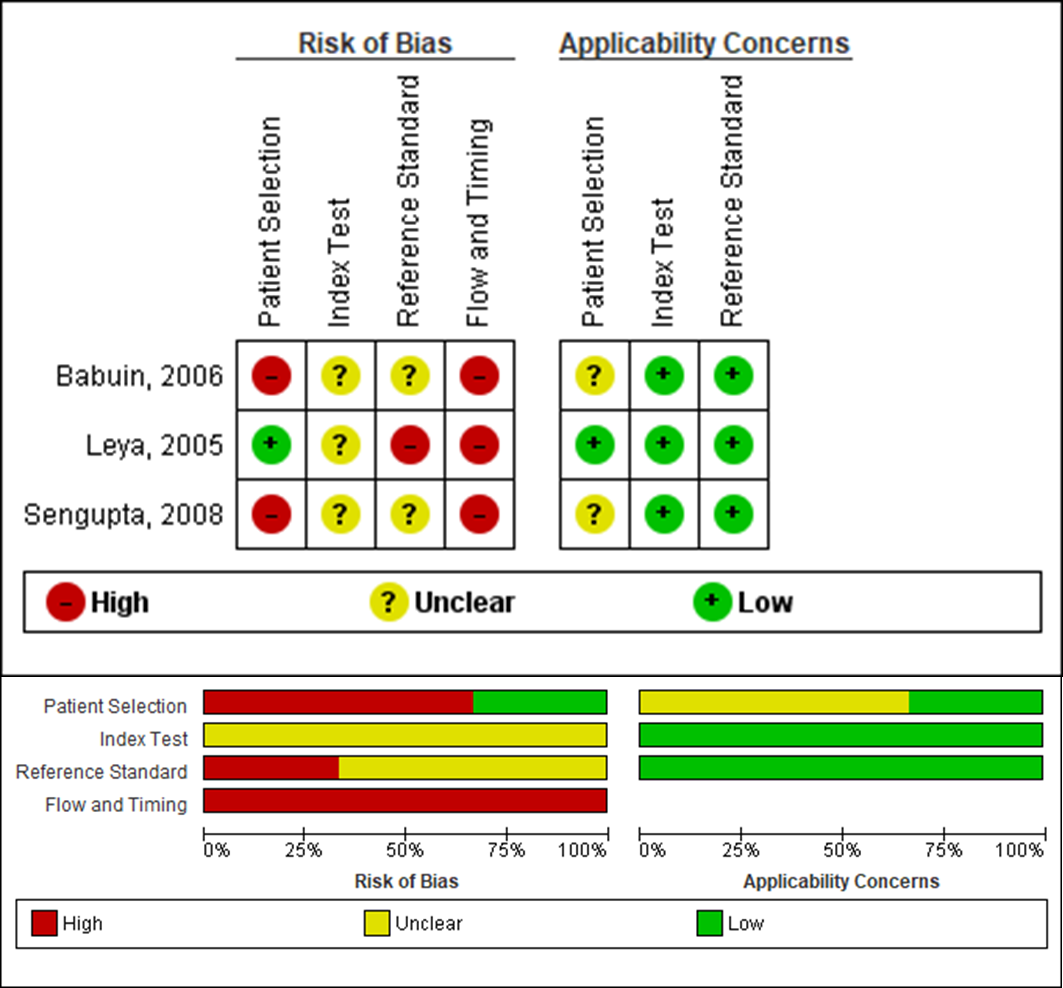
**

**Figure S1. Risk of bias and applicability concerns for each included study using QUADAS-2 tool**
